# SARS-CoV-2 and Pregnancy: A Review of the Facts

**DOI:** 10.1055/s-0040-1715137

**Published:** 2020-09-29

**Authors:** Ricardo Mamber Czeresnia, Ayssa Teles Abrao Trad, Ingrid Schwach Werneck Britto, Romulo Negrini, Marcelo Luís Nomura, Pedro Pires, Fabricio da Silva Costa, Roseli Mieko Yamamoto Nomura, Rodrigo Ruano

**Affiliations:** 1 Faculty of Medical Sciences, Santa Casa de São Paulo, São Paulo, SP, Brazil; 2 Division of Maternal-Fetal Medicine, Department of Obstetrics and Gynecology, Mayo Clinic College of Medicine, Rochester, MN, United States; 3 Department of Obstetrics & Gynecology, Hospital Israelita Albert Einstein, São Paulo, SP, Brazil; 4 Department of Obstetrics and Gynecology, School of Medical Sciences, Universidade de Campinas, Campinas, SP, Brazil; 5 Department of Gynecology and Obstetrics, School of Medical Sciences, Universidade de Pernambuco, Recife, PE, Brazil; 6 Ministério da Saúde, Brasília, DF, Brazil; 7 Department of Gynecology and Obstetrics, Faculdade de Medicina de Ribeirão Preto, Universidade de São Paulo, Ribeirão Preto, SP, Brazil; 8 Department of Obstetrics and Gynecology, Monash University, Melbourne, Australia; 9 Department of Obstetrics, Escola Paulista de Medicina, Universidade Federal de São Paulo, SP, Brazil

**Keywords:** SARS-CoV-2, pregnancy, guidelines, physiology, treatment, SARS-CoV-2, gravidez, recomendações, fisiologia, tratamento

## Abstract

**Objective** The present comprehensive review aims to show the full extent of what is known to date and provide a more thorough view on the effects of SARS-CoV2 in pregnancy.

**Methods** Between March 29 and May, 2020, the words *COVID-19* , *SARS-CoV2* , *COVID-19 and pregnancy* , *SARS-CoV2 and pregnancy* , and *SARS and pregnancy* were searched in the PubMed and Google Scholar databases; the guidelines from well-known societies and institutions (Royal College of Obstetricians and Gynaecologists [RCOG], American College of Obstetricians and Gynecologists [ACOG], International Society of Ultrasound in Obstetrics & Gynecology [ISUOG], Centers for Disease Control and Prevention [CDC], International Federation of Gynecology and Obstetrics [FIGO]) were also included.

**Conclusion** The COVID-19 outbreak resulted in a pandemic with > 3.3 million cases and 230 thousand deaths until May 2 ^nd^ . It is caused by the SARS-CoV2 virus and may lead to severe pulmonary infection and multi-organ failure. Past experiences show that unique characteristics in pregnancy make pregnant women more susceptible to complications from viral infections. Yet, this has not been reported with this new virus. There are risk factors that seem to increase morbidity in pregnancy, such as obesity (body mass index [BMI] > 35), asthma and cardiovascular disease. Current reports describe an increased rate of preterm birth and C-section. Vertical transmission is still a possibility, due to a few reported cases of neonatal positive real-time polymerase chain reaction (RT-PCR) in nasal swab, amniotic fluid, and positive immunoglobulin M (IgM) in neonatal blood. Treatments must be weighed in with caution due to the lack of quality trials that prove their effectiveness and safety during pregnancy. Medical staff must use personal protective equipment in handling SARS-CoV2 suspected or positive patients and be alert for respiratory decompensations.

## Resumo

**Objetivo** A presente revisão detalhada busca fornecer dados objetivos para avaliar o que se sabe até o momento e possibilitar uma visão mais ampla dos efeitos do SARS-CoV2 na gravidez.

**Métodos** Entre 29 de março e 2 de maio de 2020, foi realizada uma busca nos bancos de dados PubMed e Google Scholar com as palavras *COVID-19* , *SARS-CoV2* , *COVID-19 e gravidez* , *SARS-CoV2 e gravidez* , e *SARS e gravidez* . As recomendações dos principais órgãos sobre o tema também foram acessadas.

**Conclusão** O surto de COVID-19 resultou em uma pandemia com > 3.3 milhões de casos e 230 mil mortes até 2 de maio. É uma condição causada pelo vírus SARS-CoV2 e pode levar ao acometimento pulmonar difuso e à falência de múltiplos órgãos. Características únicas da gestante tornam essa população mais propensas a complicações de infecções virais. Até o momento, essa tendência não foi observada para esse novo vírus. Os fatores que parecem estar associados à maior morbidade materno-fetal são obesidade (índice de massa corporal [IMC] > 35), asma e doença cardiovascular. Há descrição de aumento de parto prematuro e parto cesáreo. Não se pode descartar a possibilidade de transmissão vertical da doença, devido a relatos de positividade de reação em cadeia de polimerase (RT-PCR) de swab nasal, RT-PCR de líquido amniótico e imunoglobulina M (IgM) de recém-nascidos. Tratamentos devem ser analisados caso a caso, dada a falta de qualidade de estudos que comprovem a sua eficácia e segurança na gravidez. O corpo clínico deve utilizar equipamentos de proteção individual (EPI) ao manusear pacientes suspeitos ou confirmados e ficar atento aos sinais de descompensação respiratória.

## Introduction

In December 2019, SARS-CoV-2 was first identified in Wuhan, China; it was the beginning of an outbreak. 1 The virus belonged to the well-known family *Coronaviridae* and mostly caused the common cold. However, this strain proved to be much more infectious than other viruses from this family such as MERS-CoV (Middle East Respiratory Syndrome) and SARS-CoV (Severe Acute Respiratory Syndrome). 1 The high rate of infectivity associated with the morbidity and mortality 2 created a world health crisis and on March 11, 2020, the World Health Organization declared a pandemic of this new disease, now named COVID-19. 3 In May 2, 2020, there were > 3.3 million confirmed cases and > 230,000 deaths caused by this outbreak. 4 Pregnancy results in unique physiological changes specifically to the immune and respiratory systems that make pregnant women more susceptible to viral infections. 5 The knowledge about the impact of SARS-CoV-2 infection in this vulnerable patient population is still limited. To address this gap, we will review the evidence available on viral characteristics, its association with pregnancy, and current management guidelines. We hope to provide resources and practical recommendations to aid in perinatal care practices.

## Methods

A search in the PubMed and Google Scholar databases was conducted daily between March 29 and May 2, 2020, with the words: *COVID-19* , *COVID-19 and pregnancy* , *SARS-CoV2 and pregnancy* , and *SARS and pregnancy* ; the guidelines focusing on pregnancy from major societies and institutions (Royal College of Obstetricians and Gynaecologists [RCOG], American College of Obstetricians and Gynecologists [ACOG], International Society of Ultrasound in Obstetrics & Gynecology [ISUOG], Centers for Disease Control and Prevention [CDC], International Federation of Gynecology and Obstetrics [FIGO]) were also reviewed. The references for the main articles were thoroughly reviewed to provide a broader comprehension of the specifics of pregnancy physiology, the pathophysiology of the virus, the main treatments, and their possible effects on pregnancy.

## Pathophysiology

Coronaviruses (CoVs) are a group of enveloped, single stranded, positive sense RNA viruses that use surface spike (S) glycoprotein on the envelope to attach host cells and mediate membrane fusion during infection. The S protein includes two regions: S1 (host cell receptor binding) and S2 (membrane fusion). For SARS-CoV, the receptor binding domain (RBD) is located in the C-Terminal Domain-1 (CTD1) of the S1 region. It has been suggested that SARS-CoV-2 infect human cells through the binding of the RBD domain to the human Angiotensin II (ACE-2) receptor; the molecular mechanism of the binding between the RBD protein and the ACE2 receptor is still unknown. 6 Two different types of SARS-CoV-2 were identified, type L (60%) and type S (30%) and we still do not know the clinical implications of this finding. 7 The transmission of SARS-CoV-2 occurs through respiratory droplets, direct contact, or fomites. Once in contact with the nasopharyngeal mucosa and pulmonary tissue, the virus enters the host-cell attaching to ACE-2 receptors and starts its replication, similar to SARS-CoV. In response, the body presents the viral antigens by antigen presenting cells (APCs) to the defense system, resulting in the production of proinflammatory cytokines and chemokines that increase the vascular permeability and lead to alveolar edema. In more severe cases, there is an overproduction of these cytokines, resulting in a cytokine storm that triggers the immune system to attack the body causing acute respiratory distress syndrome (ARDS) and multiple organ failure. 8 The CoVs have a high affinity for respiratory, enteric, hepatic, neurologic and myocardial tissues and lead to a variety of symptoms depending on the affected organ. 9 The severity of the disease can range from a common cold to severe respiratory failure, which could lead to end-organ failure and death. In the previous experiences with SARS-CoV and MERS-CoV, pregnant women had more chances of developing serious disease when compared with the general population; higher rates of adverse pregnancy outcomes (pre-eclampsia, preterm birth and fetal distress) were also identified. 10 The current evidence available on SARS-CoV-2 suggests that it does not follow this pattern, and pregnancy has not shown to increase the severity of cases. 11

### Clinical Presentation, Diagnosis, and Epidemiology

Commonly reported symptoms of COVID-19 are cough, fever, myalgia, and less frequently dyspnea, diarrhea, vomit, hemoptysis, anosmia and dysgeusia. 12 13 A systematic review by Zaigham et al 14 included 108 cases of COVID-19 in pregnancy. The main presenting symptoms were fever (68%), cough (34%), malaise (13%), dyspnea (12%) and diarrhea (6%). This presentation seems to be no different than in nonpregnant patients. 14

A suspect case is defined as the combination of symptoms and possible exposure. Currently, the gold standard test is a real-time polymerase chain reaction (RT-PCR) on respiratory samples (throat swab/ nasopharyngeal swab/ sputum), with test sensitivity estimated between 56–83%. 15 Elderly patients or those with comorbidities including diabetes, hypertension, obesity, or cardiovascular disease are at higher risk for morbidity and mortality. In a retrospective cohort, Garg et al 16 analyzed 14 US states in March 2020 and found a total of 48 hospitalized patients aged between 18 and 49 years old due to COVID-19. In this younger population, the most common comorbidities were obesity (59%), chronic pulmonary disease (36.4%), chronic metabolic disease (21.7%), and hypertension (17.5%). There were also 3 (9.9%) pregnant patients. 16

### Physiological Predisposition to Infection in Pregnancy

Unique physiological changes occur in the maternal body to allow for a healthy pregnancy. Immunologically, there are three stages in pregnancy: in the 1 ^st^ trimester, there is a complex proinflammatory chain that ensures the adequate trophoblastic invasion with no recognition of the paternal antigen; in the 2 ^nd^ stage (13 to 27 weeks), an anti-inflammatory response is necessary for adequate fetal growth and to prevent spontaneous initiation of labor; then, in the 3 ^rd^ trimester, the stimulus shifts back to a proinflammatory state for delivery. Each of these stages is a fine equilibrium that can be broken by viral infections, leading to maternal and fetal complications. 17 In theory, during the proinflammatory stages, pregnant patients would be more prone to develop cytokine storm, which is an indicator of severity in SARS-CoV-2 infection. 5 The pulmonary physiology during pregnancy suffers hormonal and functional changes that make pregnant women less tolerant to hypoxia. From the beginning of pregnancy, the levels of progesterone act on the brainstem increasing the respiratory rate and the tidal volume, the chest wall compliance decreases and so does airway resistance. 18 In the last trimester, the uterus restricts the diaphragm, which lowers the total lung capacity. 19 These respiratory adaptations associated with the immunological changes place pregnant patients at risk of developing more severe respiratory infections, as previously seen in influenza infections. 20 The hypoxemia that arises from a pulmonary infection can lead to vasoconstriction and intrauterine growth restriction (IUGR). 21

### Experience with Previous Coronaviruses during Pregnancy

There were two previous outbreaks of viruses from the *Coronaviridae* family: MERS and SARS. With little information available on SARS-CoV-2, specialists initially turned to these earlier experiences as a source of comparable data. The MERS outbreak in April 2012 lead to 2,494 confirmed cases and was responsible for 858 deaths. 22 The mortality rate was 20% amongst the population < 60 years old. Even though it belongs to the same family as SARS-CoV-2, this virus binds to a different receptor Dipeptidyl peptidase-4 (DPP4) in the alveolar cells and shares only 50% of the SARS CoV-2 genome. 23 In a retrospective study conducted in Saudi Arabia with 660 patients, there were only 11 cases of infection during pregnancy. 24 Seven (64%) of these were admitted in the ICU, there were 3 (27%) maternal deaths and 3 (27%) stillbirths; there was no evidence of vertical transmission. 24 The SARS outbreak in 2003 infected > 8,000 people and lead to 919 deaths. 25 This virus is more similar to SARS-CoV-2, sharing 80% of its genome and the same ACE-2 receptor for cell entry, although it binds with 20 times less affinity. To date, the pathophysiology is described as very similar to SARS-CoV-2, but the mortality rate was higher (11%). The pregnant population was particularly susceptible to severe forms of the disease. 26 Wong et al 27 reported on 12 cases of pregnant women in several hospitals in southern China with SARS. Seven were infected in the 1 ^st^ trimester, of which 4 had miscarriages (57%). The other 5 were infected between weeks 26 to 32; 4 had preterm births (80%) and two had fetal growth restriction (40%), there was no evidence of vertical transmission. 27

### SARS-CoV-2 Impacts on Pregnancy: Reported Cases

Based on the experience of past Coronavirus outbreaks, the unique immune response and higher morbidity associated with pulmonary infections, Liu et al 5 suggest that pregnant women might be prone to more severe forms of COVID-19. However, the available data does not support this prediction so far. The first to report COVID-19 in pregnancy, Chen et al, 28 suggested there was no increased risk of vertical transmission. But the small number of patients did not allow a definite conclusion. Breslin et al 29 did a retrospective cohort with 43 SARS-CoV-2 positive pregnant women in 3 institutions in New York; 29 (67.3%) patients presented with symptoms and the remaining 14 were asymptomatic and routinely screened before delivery. 29 Overall, 37 (86%) presented with none or mild symptoms, 4 (9.3%) had moderate symptoms and 2 (4.7%) had severe symptoms, classified with the criteria proposed by Wu et al 30 : mild disease was defined as no or mild pneumonia; severe was defined as respiratory rate > 30, SatO2 ≤ 93%, pO2/FiO2 < 300 and infiltrates > 50% of the lung; and critical disease as respiratory failure, septic shock and end-organ failure. 30 ) There was resolution of pregnancy in 18 patients, of which 10 (56%) were normal deliveries; 2 patients had worsening symptoms right after delivery, and 1, 6 days after delivery. There was only 1 preterm with 34 weeks 6/7. Nasal swab polymerase chain reaction (PCR) test for SARS-CoV-2 was negative in all tested newborns. No serology was made. Even though this study showed no evidence of increased maternal or fetal risk, they proposed screening for all pregnant women due to the high rate of initially asymptomatic cases (22.7%) It is important to point out that all the cases that presented with severe symptoms happened in women with body mass index (BMI) > 35. A systematic review by Zaigham et al 14 included 108 cases of COVID-19 in pregnancy. A total of 86 deliveries were reported, 79 (92%) cesarean and 7 (8%) vaginal deliveries. The main indication for cesarean was fetal distress, although the criteria used for this diagnosis is not clear in most studies. Lymphocytopenia was reported in 59% of the cases. The main comorbidity associated with higher morbidity was obesity. A total of two neonatal deaths were reported, both had additional risks of prematurity and cesarean. Common findings in the neonates included thrombocytopenia and lymphopenia. In this review, there were no cases with evidence of vertical transmission, although the paper points out to two case reports that described positive neonatal immunoglobulin M (IgM) and immunoglobulin G (IgG). In both cases, the nasal swab RT-PCR was negative. However, the reliability of antibody testing has been questioned since its sensitivity and specificity vary by disease, and the possibility of false-positives and cross-reactivity exists. 31 Another report described a 28-year-old obese woman with miscarriage at 19 weeks of gestation and positive PCR for SARS-CoV-2 in the placenta. No other cause for the fetal death was found, but other explanations such as spontaneous preterm birth or cervical insufficiency cannot be excluded. Even with positive PCR, fetal autopsy did not show any malformation. 32 Elshafeey et al 33 conducted a systematic review that included 385 cases of COVID-19 in pregnancy; of these, 109 (28.3%) were infected in early pregnancy. The main presenting symptoms were fever (67.3%) and cough (65.7%), and 7.5% were asymptomatic. In this review, 252 births were reported, 175 cesareans (69.4%) and 77 vaginal (30.6%). A total of 368 (95.6%) cases were classified as mild, 14 (3.6%) as severe and 3 (0.8%) were critical; 17 (4.4%) required ICU and all but 1 recovered. There were 256 newborns, 39 (15.2%) were preterm, 20 (7.8%) had low birthweight and 20 (7.8%) suffered fetal distress; 3 (1.2%) cases of neonatal death were reported. 33 It is important to mention that, on average, preterm birth rates vary from 9.3 (high income countries) to 11.8% (low income countries). 34 Regarding vertical transmission, according to Elshafeey et al, 33 4 (1.6%) had positive RT-PCR on nasopharyngeal swab, 3 (1.2%) had positive IgM, and 6 (2.3%) had positive IgG. Polymerase chain reaction for SARS-CoV2 in cord blood (30 patients), amniotic fluid (23 patients) and placenta (12 patients) were all negative. This review contains the largest number of patients so far; however, they fail to correlate the data to its original study, generating some confusion. The higher number of vaginal deliveries is expected as more cases are reported outside of China since no official guideline recommends cesarean due to SARS-CoV-2 infection. Currently, one case of maternal death due to respiratory failure from SARS-CoV-2 infection has been reported, 11 days after delivery. The same patient had positive RT-PCR for SARS-CoV-2 in the amniotic fluid and the newborn, who initially tested negative, had a positive result on the second test, 24 hours later. 35 There have been few reports on maternal complications. A case report by Gidlöf et al 36 described a pregnant woman, with BMI > 35 and pre-eclampsia bearing twins, that evolved to eclampsia after being infected by SARS-CoV-2. Additionally, Koumoutsea et al 37 brought attention to 2 cases with a possible link between 3 ^rd^ -trimester SARS-CoV-2 infection and progressive coagulopathy.

### Current Instructions on Obstetric Management: What do the Guidelines Say?

Multiple guidelines directed to health professionals in obstetric care have been published by different professional societies and international institutions. There is consensus regarding most recommendations, such as adopting telehealth when possible, use of corticosteroids if indicated even with confirmed infection, keeping route of delivery according to obstetric indications and maintenance of breastfeeding. The RCOG 38 guideline, updated on April 17, alerts for differential diagnosis that might mimic COVID-19, such as other infections and pulmonary embolism. The remaining recommendations include cardiotocography (due to the reported risk of fetal distress), isolation of all confirmed cases, use of surgical mask by SARS-CoV-2 positive patients, use of personal protective equipment by all staff and administration of low molecular weight heparin postpartum, given the low risk of hemorrhage. The use of pools during labor is contraindicated due to the risk of fecal transmission. The guideline highlights the possibility of rapid escalation of disease severity at postpartum, thus signs of deterioration such as respiratory rate > 30, saturation < 94%, FiO2 > 40% and decreased urine output must be assessed and dealt with swiftly. There is no contraindication to vaginal birth. 38 The ACOG 39 recommends screening in all suspicious cases. In case of infection in late pregnancy, postpone delivery until negative results should be attempted if no other medical indications arise. The same society recommends expedited discharge if possible (1 day for vaginal delivery and 2 days for cesarean). 39 The FIGO guidance takes into consideration the severity of the disease, dictated by symptoms and comorbidities; in suspected or confirmed cases, they recommend postpartum separation if the mother appears acutely ill and use of a dedicated breast pump instead of direct breastfeeding to avoid contact. 40 The ISUOG does not advise against the induction of labor in case of a favorable cervix, although it alerts to the lower threshold regarding fetal distress, and points out the use of negative-pressure in delivery and neonatal rooms. There is indication of chest computed tomography (CT) as a part of the workup in pregnant women. A chest CT scan may be considered as a tool for the detection of COVID-19, taking into account that it exposes the patient to a low-dose of radiation; fetal growth restriction and microcephaly have been described in high-dose exposures. The ISUOG guidance recommends that informed consent should be acquired, and a radiation shield needs to be applied over the uterus. The same institution suggests that pregnant women with suspected SARS-CoV-2 infection or with mild symptoms should be monitored with 2 to 4 weekly ultrasounds for fetal growth, amniotic fluid volume and umbilical artery Doppler if necessary. 41 The CDC recommendations regarding hospital care, last updated on April 6, determine that the number of visitors should be minimal and all should wear face masks; symptomatic women should be tested preferentially, skin to skin contact and breast-feeding in SARS-CoV-2 patients should be allowed with the use of face shield and the neonates of positive patients should be considered positive for precaution sake. 42

### Impacts of Possible Treatments in Pregnancy

The evidence on possible treatments for SARS-CoV-2 infection is still scarce. Consequently, the use of drugs needs to be taken with caution, especially in pregnancy. There have been some considerations by LaCourse et al 43 supporting the inclusion of pregnant and breastfeeding women in trials to draw more conclusions regarding this population. So far, the main pharmacotherapies described are hydroxychloroquine (HCQ), antivirals and anti-interleukines. When caring for pregnant patients, it is important to keep in mind that changes in the physiology decrease drug concentration; therefore, the dose of the medication should be adjusted accordingly. 44 Hydroxychloroquine is known to have antiviral effects; however, mechanisms found in vitro for viruses such as Chikungunya, Zika and Dengue have not been observed in vivo. 45 There has been evidence of HCQ antiviral effects against SARS-CoV-2 in vitro but there is paucity of high-quality data of these effects in vivo. 46 Although a recent clinical trial demonstrated a small benefit with the use of HCQ, issues with study design and execution bring the validity of these results into question: the unblinded and nonrandomized nature of the trial; the small number of patients included in the study ( *n* = 36); significant loss to follow-up in the intervention group ( *n* = 6); and use of a lower threshold of viral load to diagnose the disease. 47 When used in pregnant patients, there is evidence that HCQ crosses the placenta; the drug and its metabolites have been found in cord blood and neonatal urine. 48 Nevertheless, the risk posed by the drug is outweighed by the risk posed by Malaria (stillbirth, low weight at birth, premature birth) and Systematic lupus erythematosus (SLE) (pre-eclampsia, HELLP syndrome, premature birth), being classified as safe in pregnancy by the CDC. 49 To date, there is no significant evidence of the effects of SARS-CoV2 in pregnancy or of the efficacy of HCQ in the treatment of COVID-19 to justify the use of HCQ in infected pregnant women. Remdesivir inhibits RNA synthesis; it was developed for the Ebola outbreak and has been tested for other RNA viruses, such as SARS-CoV-2. Grein et al 50 conducted a multicenter study for its compassionate use in 53 severe COVID-19 hospitalized patients. Clinical improvement was observed in 36 patients (68%) and mortality was 13%. In China, the mortality rate in patients with similar conditions ranged from 22 to 66%, making these results look promising. 50 A more recent randomized, double blinded, placebo controlled study with 237 enrolled patients did not show significant reduction in overall mortality or clinical improvement. 51 During the Ebola outbreak, 6 cases of Remdesivir use in pregnant women were described and no adverse effects were reported. This course of action was only chosen due to the high mortality rate associated with Ebola, which drove the benefits of using the drug to outweigh the possible risks to pregnancy. 52 However, the number of cases is too small to draw any reliable conclusion regarding its effect on pregnancy. Tocilizumab is an anti-IL-6 monoclonal antibody. Xu et al 53 reported its use in 21 severe or critical patients and 19 (90.5%) had improvement of the overall clinical condition and were discharged. Once again, more data in less biased double-blinded randomized trials is necessary to draw any conclusion. The review by Hoeltzenbein et al 54 on 299 cases with use of Tocilizumab did not show increased risk of congenital abnormalities; there was an increased risk for stillbirth and preterm birth. There are reports of the drug crossing the placenta and of its presence in breast milk. 55

### Impacts in Mental Health

The Covid-19 outbreak is associated with adverse mental health consequences. Anxiety, depression and self-reported stress are common psychological reactions symptoms to the COVID-19 pandemic. 56 It is important that future research includes the impact on women's mental health during pregnancy and postpartum. 57

## Conclusion

The unique physiological immune state and the low tolerance to hypoxemia make pregnant women prone to more severe forms of pulmonary infections. The group was a high-risk cohort in previous Coronavirus outbreaks, such as SARS and MERS. However, this has not been observed in the COVID-19 outbreak, with symptoms and severity being described as similar to those of the general population. Whether this is because of the small number of cases that have been reported or if it reflects a unique pathophysiology of SARS-CoV2 remains uncertain. Considering the available data, pregnant women with BMI > 35, pre-eclampsia, asthma, chronic metabolic and cardiovascular disease should be managed with greater caution. In addition, patients with confirmed SARS-CoV-2 must receive special attention after delivery because of the risk of sudden worsening of pulmonary function. There is recent evidence on the possibility of vertical transmission, although there is no indication of teratogenic effects of SARS-CoV-2. The current treatment options for COVID-19 still need more research to prove efficacy and benefit; their use in pregnancy should be weighed in with caution.
